# Feasibility of stereotactic body radiation therapy with volumetric modulated arc therapy and high intensity photon beams for hepatocellular carcinoma patients

**DOI:** 10.1186/1748-717X-9-18

**Published:** 2014-01-10

**Authors:** Po-Ming Wang, Wei-Chung Hsu, Na-Na Chung, Feng-Ling Chang, Chin-Jyh Jang, Antonella Fogliata, Marta Scorsetti, Luca Cozzi

**Affiliations:** 1 Department of Radiation Oncology, Chung-Kang branch, Cheng-Ching General Hospital, Taichung, Taiwan; 2 Department of Healthcare Administration, Asia University, Taichung, Taiwan; 3 Oncology Institute of Southern Switzerland, Bellinzona, Switzerland; 4 Istituto Clinico Humanitas, Rozzano, Italy

**Keywords:** Hepatocelluar carcinoma, RapidArc, VMAT, SBRT, Flattening filter free beams

## Abstract

**Background:**

To report technical features, early outcome and toxicity of stereotactic body radiation therapy (SBRT) treatments with volumetric modulated arc therapy (RapidArc) for patients with hepatocellular carcinoma (HCC).

**Methods:**

Twenty patients (22 lesions) were prospectively enrolled in a feasibility study. Dose prescription was 50Gy in 10 fractions. Seven patients (35%) were classified as AJCC stage I-II while 13 (65%) were stages III-IV. Eighteen patients (90%) were Child-Pugh stage A, the remaining were stage B. All patients were treated with RapidArc technique with flattening filter free (FFF) photon beams of 10MV from a TrueBeam linear accelerator. Technical, dosimetric and early clinical assessment was performed to characterize treatment and its potential outcome.

**Results:**

Median age was 68 years, median initial tumor volume was 124 cm^3^ (range: 6–848). Median follow-up time was 7.4 months (range: 3–13). All patients completed treatment without interruption. Mean actuarial overall survival was of 9.6 ± 0.9 months (95%C.L. 7.8-11.4), median survival was not reached; complete response was observed in 8/22 (36.4%) lesions; partial response in 7/22 (31.8%), stable disease in 6/22 (27.3%), 1/22 (4.4%) showed progression. Toxicity was mild with only 1 case of grade 3 RILD and all other types were not greater than grade 2. Concerning dosimetric data, Paddick conformity index was 0.98 ± 0.02; gradient index was 3.82 ± 0.93; V_95%_ to the clinical target volume was 93.6 ± 7.7%. Mean dose to kidneys resulted lower than 3.0Gy; mean dose to stomach 4.5 ± 3.0Gy; D_1cm_^3^ to spinal cord was 8.2 ± 4.5Gy; D_1%_ to the esophagus was 10.2 ± 9.7Gy. Average beam on time resulted 0.7 ± 0.2 minutes (range: 0.4-1.4) with the delivery of an average of 4.4 partial arcs (range: 3–6) of those 86% non-coplanar.

**Conclusions:**

Clinical results could suggest to introduce VMAT-RapidArc as an appropriate SBRT technique for patients with HCC in view of a prospective dose escalation trial.

## Background

Hepatocellular carcinoma (HCC) is the third cause of cancer death and one of the most challenging oncological problems [1]. Several treatment techniques, including surgery, transcatheter arterial chemoembolization, radiofrequency ablation, percutaneous ethanol injection, chemotherapy and targeted agents have been explored with complex decision trees and limited impact on outcome [2-6].

Conventional radiotherapy was offered to HCC patients in the past but it was limited by severe radiation induced liver disease (RILD) when excessive fractions of the liver were involved in the radiation field [7] and the important relationship between the volume of irradiated normal liver and the toxicity profile [8,9]. Recently, volumetric modulated arc therapy (VMAT) was explored in the frame of investigating the role of intensity modulation with rotational techniques. Our previous studies [10,11] reported about treatment of 138 HCC patients showing 1-year and 2-year survival rates around 80-100% and 60-100% for stage I-II patients, respectively.

Stereotactic body radiation therapy (SBRT) approaches have been hypothesized for smaller target and in cases requiring modest irradiation of entire liver (less than 1/4 of the volume). Tse et al. [12] reported on a phase-I liver cancer dose-escalation trial which included 31 HCC patients. With a median dose 36 Gy in 6 fractions, they achieved a 12 month local control rate of 65%. Overall survival was 48% at one-year. Kwon et al. [13] reported about 42 HCC patients treated with a median dose of 33 Gy in 3 fractions and a local control of 72 and 67.5% at 1 and 2 years, respectively. Consistent with other SBRT studies, their result had very low toxicity (less than 2%) and low incidence of RILD (2%). Andolino et al. [14] summarized the treatment results of 60 patients, Child-Pugh (CP) stage A, received 30–48 Gy in 3 fractions. Local control was 93% at 1-year with a 1-year overall survival of 93% (77% at 2 years). CP stage B patients received 24–48 Gy in 3 fractions with a relative lower survival (70% at 1 year). Bujold et al. [15], from a cohort of 102 patients treated in two sequential trials between 2004 and 2010, showed a 1 year local control of 87% with a clear association of better outcome with higher SBRT doses, although the toxicity higher than grade 3 was observed in one third of the patients (a severe possibly treatment related fatalities were observed as well). Klein and Dawson [16] concluded that SBRT in HCC can have comparable results with other therapies and suggested that it might be offered as an treatment option for early stage HCC patients or patients not eligible to other ablative procedures for any reason.

In general, there is still no systematic large scale trial performed to assess i) best treatment modality for SBRT from technical point of view and ii) maximum tolerable dose in absence of severe complications. In this frame, the present study assessed as a first step on a group of prospectively treated patients, the feasibility of a moderate SBRT regimen delivering 50Gy in 10 fractions with RapidArc using high intensity photon beams (flattening filter free (FFF) beams). Scope was to determine the feasibility and the safety of the treatment as a preliminary component to a formal dose escalation trial to be started at the home institute with a more aggressive fractionation scheme (3 fractions with dose levels from 12Gy up to the maximum tolerable dose, not superior to 25Gy per fraction).

## Methods

### Patients

Starting from September 2012, 20 HCC patients presented with Barcelona Clinic Liver Cancer (BCLC) stage A to C (one stage 0) and CP stages A-B with single or double lesions (preferably with tumor size >5 cm or because of the poor liver reserves) were eligible for SBRT with moderate hypofractionation and were enrolled in a prospective feasibility study at the home institute. The institute’s ethical committee approved the protocol. All patients received radiotherapy as primary treatment and at least 30 elapsed days after any prior treatment. Portal vein thrombosis was present in six (35%) patients. Relative contraindication to inclusion were: total bilirubin levels greater than 3 to 5 mg/dL; white blood count (WBC) less than 2500–1500 U/dL; Glutamic pyruvic transaminase (GPT) in the range of 100–300 U/L. Absolute exclusion criteria included total bilirubin >5 mg/dL, WBC < 1500 U/dL and GPT > 300 U/L. Patients violating relative contraindications were enrolled on a case-by-case based on medical decision.

### Radiation treatment

Dose prescription was of 50Gy in 10 fractions, 5Gy/fraction (this is the lower level dose prescription in a risk adapted scheme; lesion with a size between 3 and 5 cm would receive 8-12Gy x 5 fractions and patients with lesions <3 cm would be treated with 15-25Gy x 3 fractions; all depending on tumor location and normal tissue tolerance). Plans were designed for single course treatments. The gross tumor volume (GTV) was defined as the primary tumor plus abnormal portal areas revealed on 4 dimensional gated computerized tomography (4D-CT) images, intravenous contrast medium administration for enhancement was used for all patients, except for those patients with renal dysfunction, Magnetic resonance image (MRI) was added and fused to planning CT for more precise target delineation. The clinical target volume (CTV) was defined as the GTV. For all patients, an internal target volume (ITV) was defined as the envelope of all CTVs from the different respiratory phases and used for treatment planning as the equivalent of PTV. Treatment planning was performed on the end-expiration phase.

Plans were optimized aiming to a CTV coverage: V_95%_ > 90%, (ITV: V_90%_ > 90%) and mean CTV (ITV) dose >50Gy. For all patients, in addition to the target volume, the entire liver, the normal liver (whole liver-PTV), the kidneys, the stomach, the spinal cord were outlined and considered during optimization. The following explicit planning objectives were defined: for the total liver V_30Gy_ < 60%, for the normal liver (liver-ITV), the volume receiving less than 15Gy was asked to be greater than 700 cm^3^. For the stomach V_37.5Gy_ < 5%, for the kidneys: V_15Gy_ < 35%, for the spinal cord: D_1cm_^3^ < 22Gy.

All patients were treated by VMAT in the form of RapidArc with 10 MV FFF photon beam generated by a Varian TrueBeam linear accelerator, which equipped with the high definition MLC (2.5 mm of leaf resolution at isocenter in the central 20 cm). Maximum allowed dose rate was set to 2400MU/minute. Individualized optimization was performed using single or multiple, coplanar or non-coplanar, mono-isocentric arcs.

All patients were treated in supine position with arms placed overhead and were immobilized with individualized masks and a vacuum cushion for positioning. Delivery was performed with respiratory gated RapidArc to treat only the selected respiratory phase (end-expiration with a duty cycle of 30-70%). Image guidance during treatment was exploited by means of daily cone-beam CT acquisition to verify in 3D the proper positioning of patients.

### Evaluation

Dosimetric and technical parameters of delivery were scored including some delivery parameters as well as standard analysis of dose volume histograms (DVH). Homogeneity was scored as HI = V_5%_-V_95%_)/D_mean_ as well as in terms of standard deviation. Paddick Conformity and Gradient indexes (PCI and PGI) were defined and reported [17,18]. PCI = TV_PIV_^2^/(TV_PIV_ x PIV) where TV_PIV_ is the target volume irradiated at prescription dose and PIV is the prescribed isodose volume; PGI = V_50%PIV_/PIV where V_50%PIV_ is the volume irradiated at 50% of the prescribed dose. Clinical evaluation was performed after treatment completion and visits included laboratory assessment and CT and/or MRI imaging, with reference to baseline conditions determined before start of treatment, during treatment, at 1, 2, 3,6 months after treatment completion, and then every three-month interval. For basic treatment outcome was measured in terms of in-field local control. Tumor response was assessed using modified Response Evaluation Criteria in Solid Tumors (mRECIST) criteria for HCC [19]. Local in field recurrence was defined by new enhancement or progressive disease with CT or MR imaging during follow-up. RILD was defined as elevated transaminase of at least two-fold the upper limit of normal value or pretreatment levels based on the National Cancer Institute Common Toxicity Criteria for Adverse Events (CTCAE) version 4.03. Endpoints of gastrointestinal (GI) toxicity included esophagitis, gastritis, gastric hemorrhage or ulceration, duodenal hemorrhage or ulceration and ascites were also scored according to CTCAE 4.03. Statistical analysis was performed by means of the SPSS package (version 20, IBM). Survival was determined by Kaplan-Meier methods and reported stratified according to several variables. Log-rank tests were applied to estimate significance of differences among survival curves.

## Results

### Patients

A total of 22 liver lesions were treated in the group of 20 patients. Table 1 summarizes the characteristics of the cohort of patients included in the study. For all patients, median initial tumor volume was 124 cm^3^ (ranging from 6 to 848 cm^3^) corresponding to <10% of the median total liver volume. Patients presented with uniform distribution of BCLC (0-A, B or C) or AJCC (I-II, III and IV) stages. For 3 patients with nodal involvement (1 in porta hepatis, 1 in para-aortic area and 1 in right cardiophrenic angle) and 3 with distant metastasis (1 in right upper lung, 1 in right 8th rib, and 1 in peritoneum), the irradiation was limited to the liver localisaton and and no chemotherapy or other systemic therapy was combined concurrently. The vast majority of patients presented with CP stage A (90%); the majority had no portal vein thrombosis. Hepatitis B had the most frequent incidence although 25% of patients had no hepatitis at diagnosis. One patient presented with both N1 M1 stages, 2 patients with N1 and 2 with M1 stages. These patients were treated as discussed for their primary HCC localisation.

**Table 1:** Characteristics of the cohort of patients

**Characteristics**	**Items**	**N (%)**
**Sex**	Female	8/20 (40%)
	Male	12/20 (60%)
**Age**	Mean	64.5
	Median (range)	68.55 (47–81)
	St.dev	11.2
**Portal vein thrombosis**	No	14/20 (65%)
	Yes	6/20 (35%)
**Tumor location**	Right lobe	7/22 (32%)
	Left lobe	4/22 (18%)
	Bilateral lobe	11/22 (50%)
**Stage T**	T1	3/20 (15%)
	T2	4/20 (20%)
	T3	12/20 (60%)
	T4	1/20 (5%)
**Stage N**	N0	17/20 (85%)
	N1	3/20 (15%)
**Stage M**	M0	17/20 (85%)
	M1	3/20 (15%)
**AJCC stage**	I	3/20 (15%)
	II	4/20 (20%)
	III	8/20 (40%)
	IV	5/20 (25%)
**Okuda stage**	I	16/20 (80%)
	II	4/20 (20%)
**BCLC stage**	0	1 (5%)
	A	3 (15%)
	B	8 (40%)
	C	8 (40%)
**Child-pugh stage**	A	18 (90%)
	B	2 (10%)
**Hepatitis**	No	5 (25%)
	B	10 (50%)
	C	5 (25%)
	B and C	0 (0%)
**Initial Alpha-fetoprotein (ng/mL)**	Median (range)	301.4 (2.8 – >52131)
**Initial white blood count (kU/dL)**	Median (range)	5.2 (3.3 – 10.7)
**Initial haemoglobin level (g/dL)**	Median (range)	12.0 (6.7 – 15.5)
**Initial GPT level (U/L)**	Median (range)	41.5 (18.0 – 244.0)
**Initial total bilirubin level (mg/dL)**	Median (range)	0.8 (0.3 – 1.9)
**Initial tumor volume (cm**^**3**^**)**	Median (range)	124 (6 – 848)
**Total liver volume (cm**^**3**^**)**	Median (range)	1343 (928 – 2198)

AJCC: American joint Committee on Cancer, BCLC: Barcelona Clinic Liver Cancer. HCC: hepatocellular carcinoma. GPT: Glutamic Pyruvic Transaminase.

Values refer to number of patients, % to the total number of 20 patients.

### Treatment

Table 2 shows the summary of the technical delivery parameters of the Rapidarc plans. Multiple non-coplanar arcs were used in 86% of the cases with relatively short individual arc length (in average <80 degrees per arc). Due to the use of FFF beams and high dose rates (on average about 60% of the maximum allowed), the nominal beam on time (BOT) per fraction resulted very short (<1 minute in average). The actual time shall be increased, due to the respiratory gating duty cycle of about 40% resulting in average of 1.8 minutes.

**Table 2:** Summary of technical delivery parameters

**Items**	**N (%)/Mean ± SD (range)**
**Number of partial arcs**	4.4 ± 0.9 (3–6)
**Coplanar arcs**	Yes: 3 (14%)
	No: 19 (86%)
**Arc length (deg)**	76.6 ± 55.5 (30;240)
**MU/Gy**	190 ± 46 (136;351)
**Dose rate (MU/minute)**	1454 ± 382 (922–2400)
**BOT (min)**	0.7 ± 0.2 (0.4;1.4)
**CP area (cm**^**2**^**)**	59.5 ± 42.9 (8.2;192.2)
**Collimator angle**	30.8 ± 12.4 (13;48)

CP = Control Point; BOT = Beam on time, BOT = beam on time, MU = Monito Units.

SD: standard deviation.

**Figure 1:**
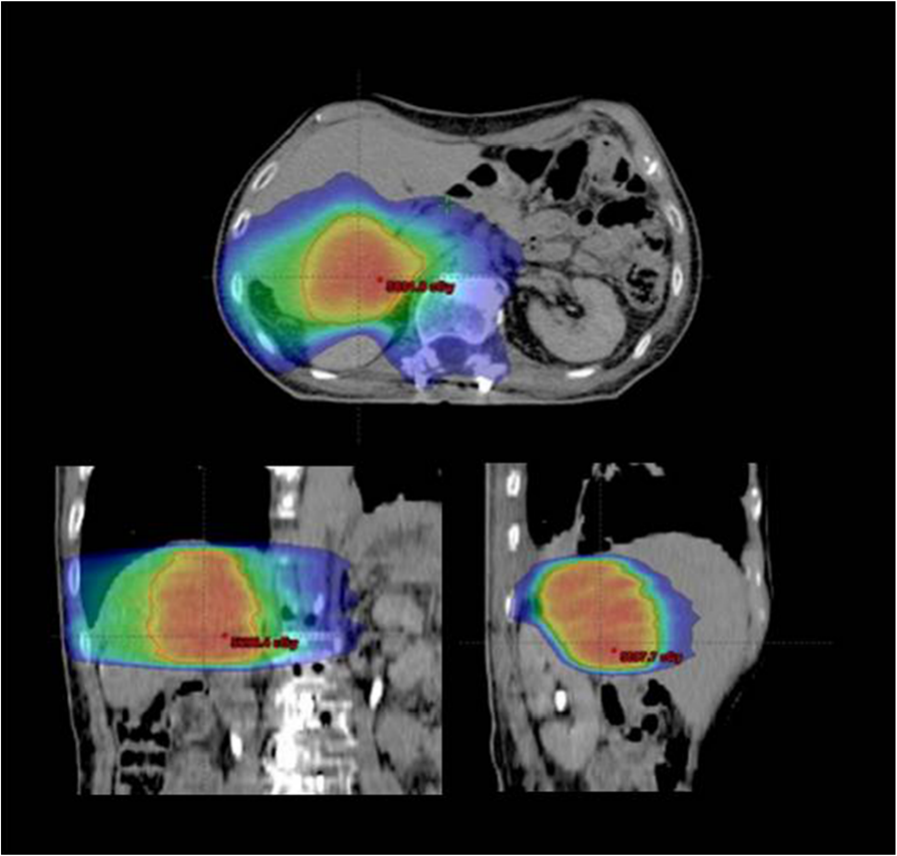
**Isodose distributions for a typical patient in coronal, sagittal and axial planes.** Color-wash threshold was set to 15Gy.

**Figure 2:**
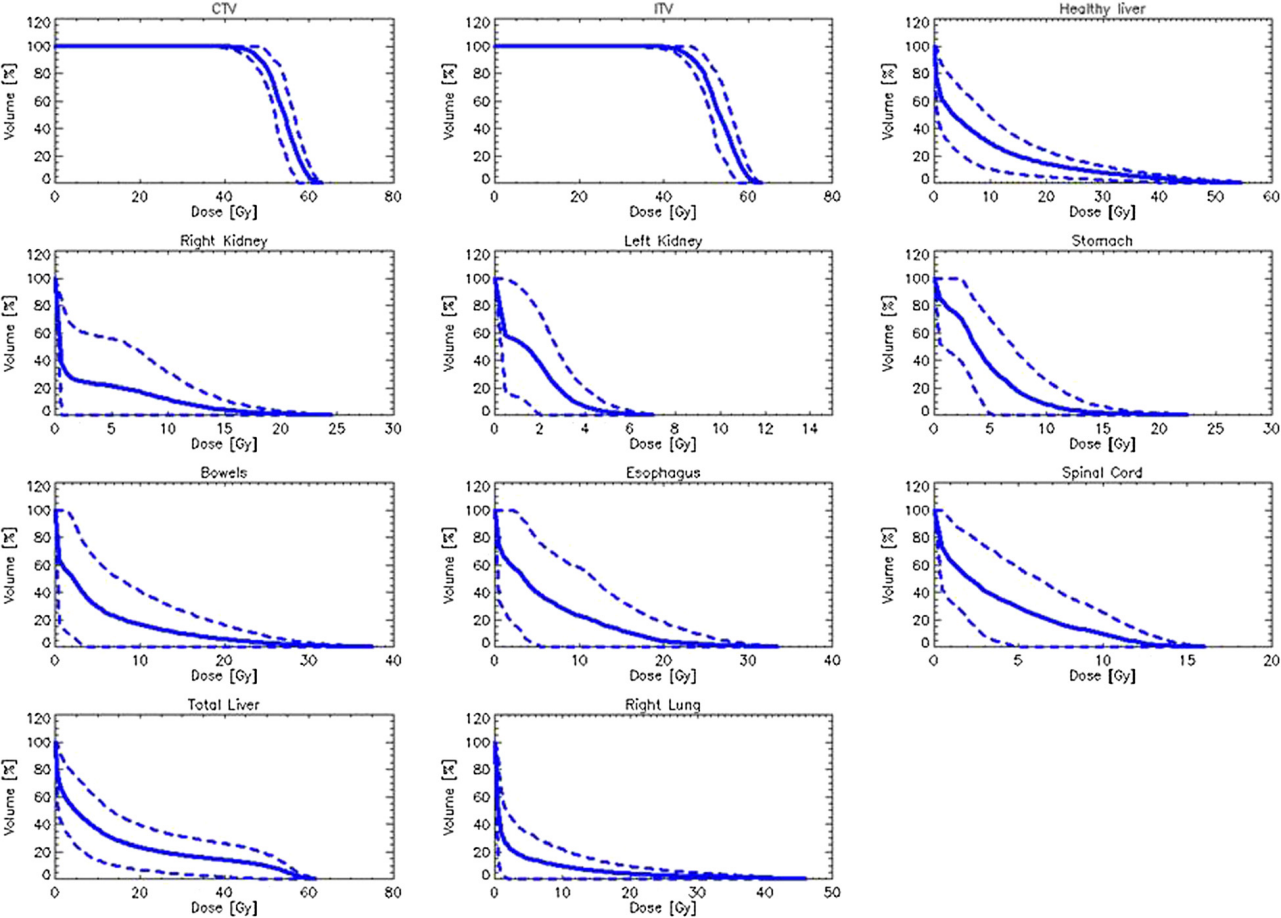
**Average dose volume histograms for CTV, ITV and organs at risk (solid lines).** Dashed lines represent inter-patient variability at one standard deviation.

Table 3 shows results from the dose volume histogram (DVH) analysis for CTV and ITV; Table 4 reports the same for the organs at risk (OARs). Figure 1 shows a typical dose distribution for one patient with the color-wash of dose distribution set to 15Gy. Figure 2 shows the average dose volume histograms (solid lines) for the target volumes and the OARs. Dashed lines represent inter-patient variability at 1 standard deviation. The coverage requirement on CTV and ITV was on average respected as well as the constraint on mean target dose. Conformity resulted almost optimal (ideal PCI = 1) while, due also to the relatively large size of the targets, dose gradient was not pushed at maximum during optimization resulting in an average value slightly inferior to 4. High target homogeneity was on the contrary easily achieved resulting in a standard deviation to CTV or ITV <8% of the prescription dose or HI ~ 0.2. The planning objectives for OARs were in general respected as well. For kidneys, V_15Gy_ resulted identically 0 in all cases and the average near-to-maximum dose (D_1cm_^3^) ranged from ~3.5Gy for the contra-lateral organ to ~11Gy for the ipsilateral one. Similarly for the stomach, no patient exceeded the threshold set to V_37.5Gy_ and the near-to-maximum dose was kept below 10Gy. D1cm^3^ to spinal cord resulted ~8Gy, largely inferior to the objective constraint of 22Gy. V_30Gy_ for total liver resulted ~14%, i.e. with a further 46% sparing compared to the planning objective. Concerning normal liver (liver-ITV), the volume receiving less than 15Gy resulted on average near to 1000 cm^3^ with only one patient marginally violating the planning objective (668 cm^3^ instead of at least 700 cm^3^).

**Table 3:** Summary of the DVH analysis for the CTV and ITV for the entire cohort of patients

**Parameter**	**CTV**	**ITV**
**Volume (cm**^**3**^**)**	131.1 ± 85.2	166.2 ± 200.2
**Mean (Gy)**	53.8 ± 2.7	53.1 ± 2.3
**SD (Gy)**	3.3 ± 1.0	3.9 ± 1.1
**HI (Gy)**	0.19 ± 0.03	0.22 ± 0.05
**PCI**	n.a.	0.98 ± 0.02
**PGI**	n.a.	3.82 ± 0.93
**D**_**1% **_**(Gy)**	58.7 ± 3.1	58.5 ± 3.4
**D**_**99% **_**(Gy)**	45.0 ± 3.7	42.7 ± 3.9
**V**_**90% **_**(%)**	97.6 ± 4.1	94.8 ± 5.4
**V**_**95% **_**(%)**	93.6 ± 7.7	89.5 ± 10.0

St. Dev: standard deviation; Dx%: dose received by at least x% of the volume; Vx%: volume receiving at least x% of the dose. HI = homogeneity index, PCI = Paddick conformity index, PGI = Paddick gradient index, n.a, not applicable.

Data are reported as average values plus or minus standard deviation.

**Table 4:** Summary of the DVH analysis for the organs at risk for the entire cohort of patients

**Organ**	**Volume (cm**^**3**^**)**	**Parameter**	**Mean ± SD**	**Range**
**Left kidney**	174.2 ± 51.7 (123.2;218.7)	Mean (Gy)	1.5 ± 1.3	(0.1;3.3)
		D_1cm3_ (Gy)	3.5 ± 3.1	(0.1;6.9)
		V_15Gy_ (%)	0.0 ± 0.0	(0.0;0.0)
**Right kidney**	182.3 ± 37.8 (135.8;213.1)	Mean (Gy)	2.8 ± 4.1	(0.1;8.8)
		D_1cm3_ (Gy)	10.6 ± 12.3	(0.2;25.1)
		V_15Gy_ (%)	4.1 ± 7.2	(0.0;14.8)
**Spine**	22.7 ± 7.2 (9.9;35.6)	D_1cm3_ (Gy)	8.2 ± 4.5	(1.9;16.7)
**Stomach**	174.8 ± 117.2 (76.3;473.2)	Mean (Gy)	4.5 ± 3.0	(3.0;36.6)
		D_1cm3_ (Gy)	9.2 ± 5.8	(0.3;22.4)
		V_37.5Gy_ (%)	0.0 ± 0.0	(0.0;0.0)
**Esophagus**	10.3 ± 6.3 (4.8;21.7)	Mean (Gy)	5.9 ± 7.9	(0.1;19.4)
		D_1%_ (Gy)	10.2 ± 9.7	(0.1;30.1)
		V_50Gy_ (%)	0.0 ± 0.0	(0.0;0.0)
**Liver-PTV**	1242 ± 330 (780;1981)	Mean (Gy)	8.6 ± 4.6	(1.4;17.1)
**(Healthy liver)**				
		V < 15Gy (cm3)	995.2 ± 163.6	(667.6;1718.2)
		V_30Gy_ (%)	24.4 ± 11.6	(0.6;56.4)
**Total liver**	1439 ± 386 (928;2197)	Mean (Gy)	13.3 ± 8.3	(2.7;32.9)
		V_30Gy_ (%)	13.8 ± 12.1	(1.1;49.3)
**Healthy tissue**	20512 ± 5059 (11277;28676)	Mean (Gy)	2.5 ± 1.7	(0.5;7.3)
		V_10Gy_ (%)	6.1 ± 4.6	(0.9;19.5)
		DoseInt (Gy*cm^3^*10^4^)	6.3 ± 2.7	(0.1;14.2)

Dx%: dose received by at least x% of the volume; Vx%: volume receiving at least x% of the dose; DoseInt: integral dose.

Data are reported as average values plus or minus standard deviation and range.

### Response and toxicity

**Figure 3:**
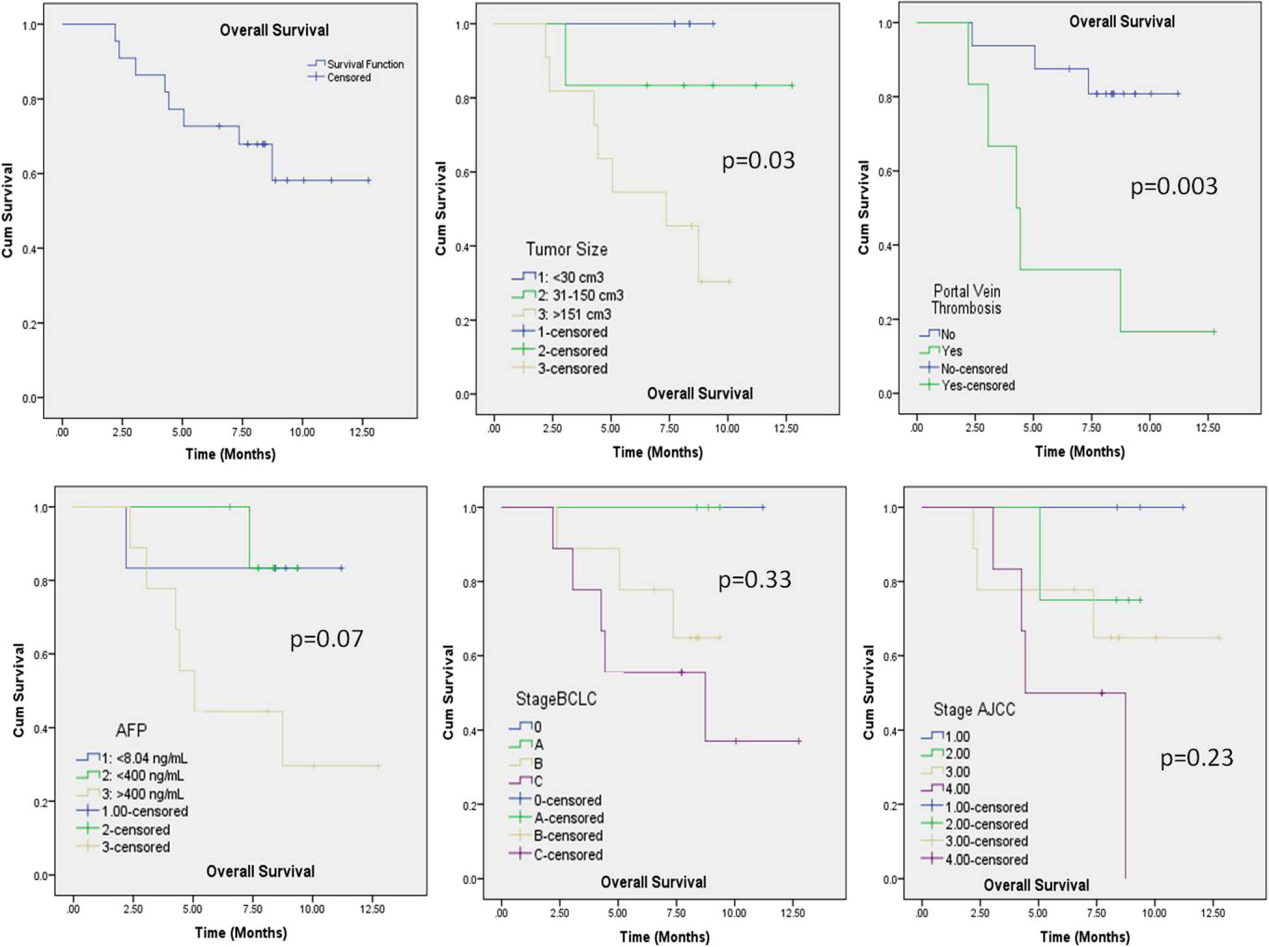
Kaplan-Meier survival curves for the entire cohort of patients and stratified according to tumor size, presence of portal vein thrombosis, initial AFP level, AJCC and BCLC stage.

**Figure 4:**
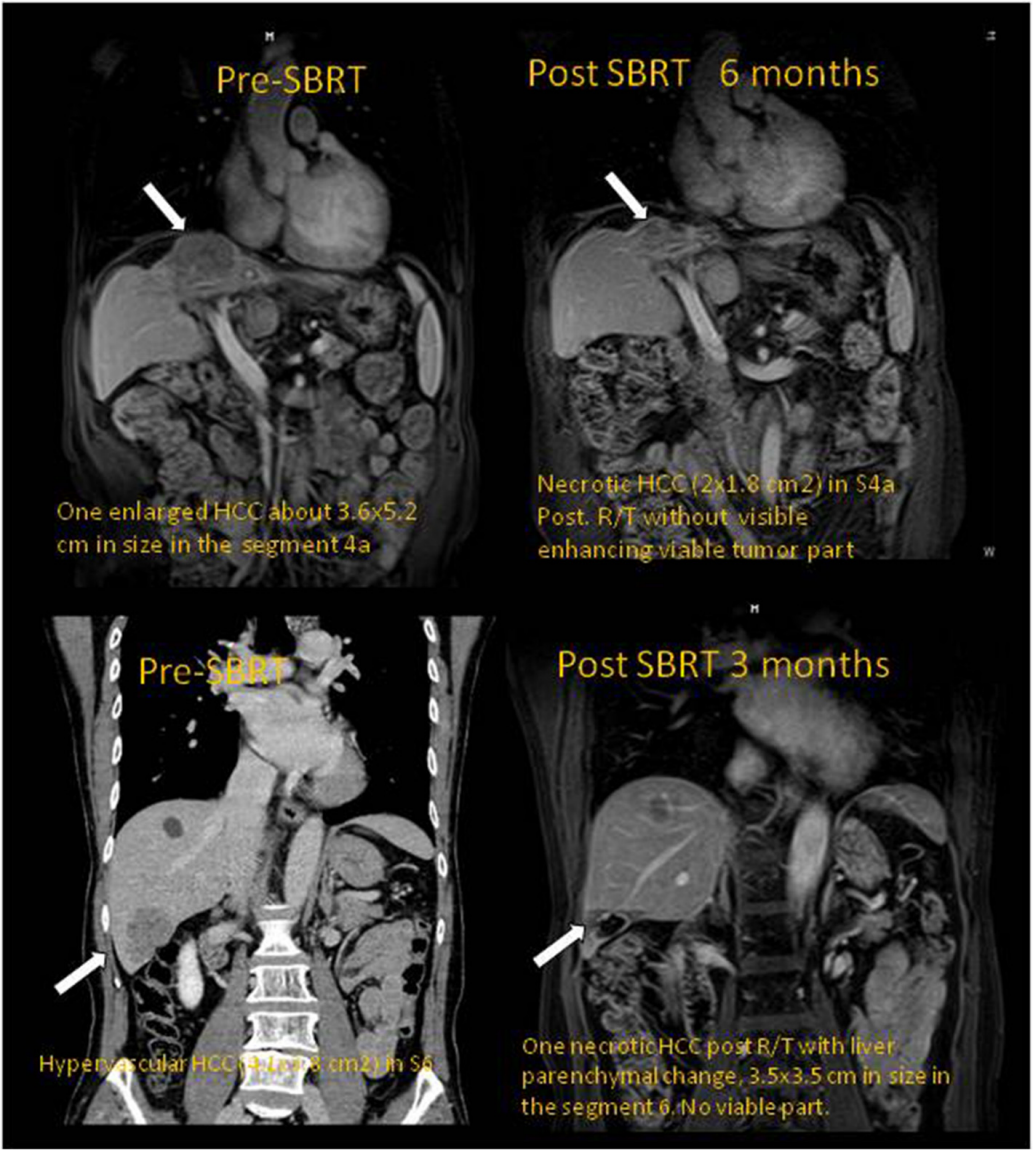
**The radiological response for two patients at MR at 6 and 3 months after treatment.** In both cases, a residual necrotic mass was detected in the position of the primary HCC without visible enhancing of any viable tumor residual.

The median follow up was 7.4 months (range: 3–13). Mean actuarial overall survival was of 9.6 ± 0.9 months (95%C.L. 7.8-11.4 months), median survival was not yet reached. Figure 3 shows Kaplan-Meier curves for overall survival for the whole cohort of patients and factorized for some risk factors. Only initial tumor size and presence of portal vein thrombosis lead to significant differences in survival. Initial Alpha-fetoprotein (AFP) level showed a near-to-significance trend. Complete response was observed in 8/22 (36.4%) lesions; partial response in 7/22 (31.8%), stable disease in 6/22 (27.3%), 1/22 (4.4%) showed progression. Figure 4 shows the radiological response for two patients at MR (6 and 3 months after treatment). In both cases, a residual necrotic mass was detected without visible enhancing of any viable tumor residual

**Figure 5:**
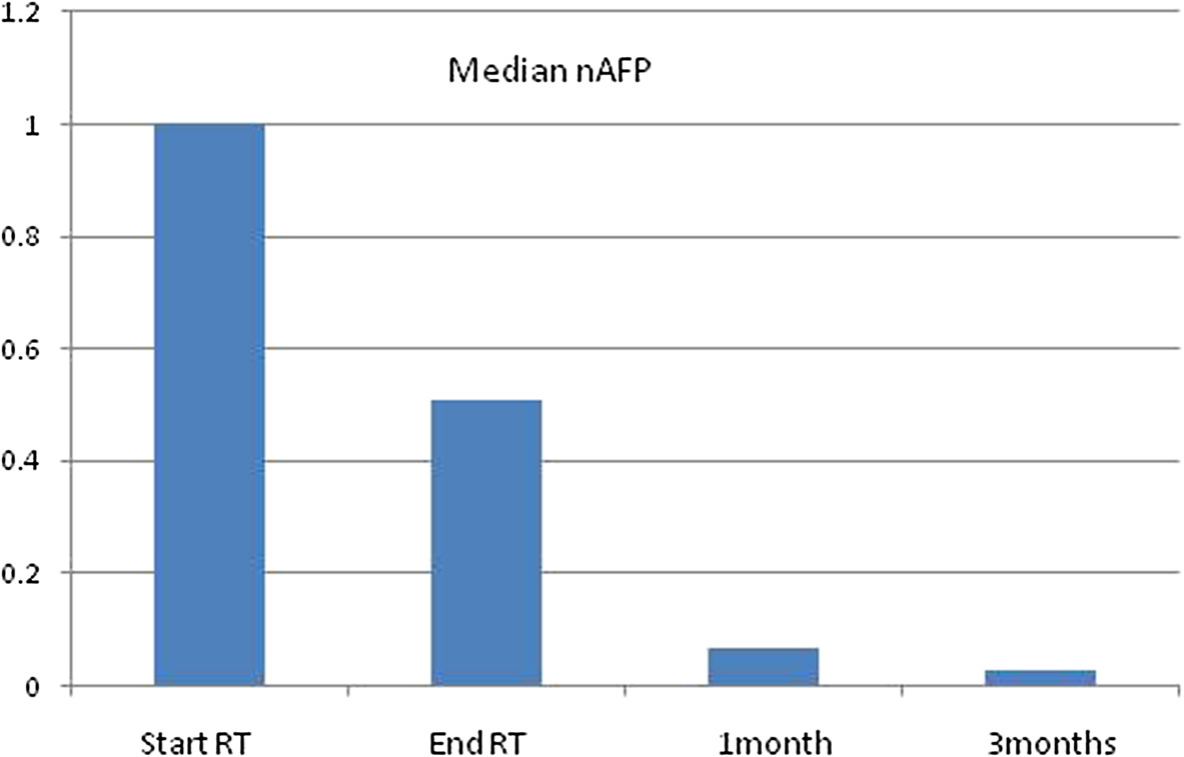
The reduction of the median nAFP (AFP normalized to the baseline) over time.

Alpha-fetoprotein (AFP) level was monitored and its reduction from baseline before treatment to the end of radiotherapy and during follow-up resulted highly significant for the entire cohort of patients. Figure 5 shows the reduction of the median nAFP (AFP normalized to the baseline) over time. At end of therapy, nAFP dropped to 50% of the baseline; at 3 months after treatment completion n AFP was only 7% of the baseline (p < 0.01 in both cases). Different trends were observed for the two risk groups (baseline AFP between normal level to 400 ng/L versus greater than 400 ng/L). For the first risk group, AFP remained either stable or presented a mild increase during treatment and follow-up. The patients belonging to the highest risk group, showed, on the contrary, the most remarkable reduction of AFP.

No impact was observed on total bilirubin levels between baseline (0.75 ± 0.40 mg/dL, range: 0.30-1.90) and finding at 6 months (0.85 ± 0.50 mg/dL, range: 0.50-1.60) and end of therapy.

A significant (p = 0.01) drop in WBC was observed between baseline and end of therapy (5.2 ± 2.1 vs 4.4 ± 1.8 U/dL), this was recovered at 3 months after treatment completion. No trends were observed for hemoglobin levels.

Concerning treatment toxicity, non-classical RILD, including ascites, resulted the most common in 7/20 patients (35%) with only one case of grade 3. No esophagitis, gastric hemorrhage or duodenum ulcer was observed. Remaining toxicities did not exceed the grade 2. GI toxicity is summarized in Table 5.

**Table 5:** Summary of toxicity profiles based on CTCAE 4.03 criteria

	**Gr 1**	**Gr 2**	**Gr 3**	**Gr 4**
RILD	1 (5%)	5 (25%)	1 (5%)	0
Esophagitis	0	0	0	0
Gastric hemorrhage	0	0	0	0
Gastric ulcer	0	2 (10%)	0	0
Gastritis	0	4 (20%)	0	0
Duodenum hemorrhage	0	3 (15%)	0	0
Duodenum ulcer	0	0	0	0
Ascites	2 (10%)	4 (20%)	0	0

## Discussion

A prospective study was carried out to assess technical feasibility and basic safety of SBRT for treatment of HCC patients in view of a phase I trial for the determination of maximum tolerable dose in early stage patients. For the current study, a small cohort of patients with different tumor stages with lesions greater than 5 cm, was enrolled and treated with RapidArc using FFF beams and high dose rate. A moderate hypofractionation regimen of 5Gy/fraction was adopted because of the size of primary lesions; more aggressive scheduling is foreseen for the forthcoming trial. The present study is a feasibility-follow-up of the previous institutional experience where a cohort of 138 patients were retrospectively analyzed after treatment with RapidArc with conventional fractionation showing promising results in terms of local control and survival [10,11]. This investigation demonstrated that hypofractionated treatments are technically feasible with the possibility to easily respect planning constraints for OARs and target volumes. Results demonstrated the possibility to treat with SBRT these HCC patients using RapidArc safely and effectively with minimum incidence of acute toxicity. Local control results are in line with earlier data. A significant clinical effect at biochemical level was observed in the decline of the AFP levels at the end of treatment and during follow-up.

The optimisation of treatment plans was performed using most frequently non-coplanar arcs and with this feature, RapidArc allowed to adequately spare normal liver tissue. To account for the internal organ motion and to establish the needed expertise for the future, all deliveries were performed with respiratory gating. RapidArc has been technically proven to be un-effected by gating [20,21] and in the present study patients were treated in free breathing with phase-based gating at end-expiration (breath hold or deep inspiration are impractical for the patients with huge HCC and massive ascites). The significant increase in treatment time due to the low duty cycle (~40%) was largely compensated by the fast nominal beam on time derived from the RapidArc technique itself and the usage of FFF beams with a maximum dose rate of 2400MU/minute. As the study of Scorsetti et al. [22] stated, in practice the average dose rate reached only ~50% of its potential suggesting that treatment time will not be an issue also when higher dose per fractions should be applied. For this reason, also deep inspiration breath hold might be investigated as well, for at least some subset of patients capable to cope with it, if associated to FFF beams.

## Conclusion

Early results in terms of local control and toxicity for patients treated with RapidArc under moderated SBRT fractionation regimen, demonstrated the appropriateness of this technique and its feasibility for SBRT in primary liver cancer. Longer follow-up will allow to consolidate these early observations. Dose escalation for early stage patients will be explored in a separate prospective trial.

## Consent

Written informed consent was obtained from the patient for the publication of this report and any accompanying images.

## Competing interests

Dr. L. Cozzi is Head of Research at Oncology Institute of Southern Switzerland and acts as Scientific Advisor to Varian Medical Systems.

## Authors’ contributions

PMW and LC coordinated the entire study. Data collection was conducted by PMW, WCH, NNC, FLC. Data were analyzed by PMW, WCH, NNC, FLC, CJJ, AF, LC. All authors read and approved the final manuscript.
